# Artemisinin Binds and Inhibits the Activity of *Plasmodium falciparum* Ddi1, a Retroviral Aspartyl Protease

**DOI:** 10.3390/pathogens10111465

**Published:** 2021-11-11

**Authors:** Noah Machuki Onchieku, Sonam Kumari, Rajan Pandey, Vaibhav Sharma, Mohit Kumar, Arunaditya Deshmukh, Inderjeet Kaur, Asif Mohmmed, Dinesh Gupta, Daniel Kiboi, Naseem Gaur, Pawan Malhotra

**Affiliations:** 1 Malaria Biology Group, International Centre for Genetic Engineering and Biotechnology, New Delhi 110067, India or noahmachuki@yahoo.com (N.M.O.); vaibhavhotrod@gmail.com (V.S.); arunaditya1@gmail.com (A.D.); inderjeet.bachhal@gmail.com (I.K.); 2 Yeast Biofuel Group, International Centre for Genetic Engineering and Biotechnology, New Delhi 110067, India; sonamverma.sv85@gmail.com (S.K.); mohitplawat007@gmail.com (M.K.); naseem@icgeb.res.in (N.G.); 3 Translational Bioinformatics Group, International Centre for Genetic Engineering and Biotechnology, New Delhi 110067, India; rajan.pandey1990@gmail.com (R.P.); dinesh@icgeb.res.in (D.G.); 4 Parasite Cell Biology Group, International Centre for Genetic Engineering and Biotechnology, New Delhi 110067, India; amohd@icgeb.res.in; 5 Department of Biochemistry, Jomo Kenyatta University of Agriculture and Technology, Nairobi P.O. Box 62000-00200, Kenya; muthuikiboi@gmail.com

**Keywords:** artemisinin, *Plasmodium falciparum*, DNA damage, Ddi1, ubiquitin-proteasome pathway, enzyme inhibition

## Abstract

Reduced sensitivity of the human malaria parasite, *Plasmodium falciparum,* to Artemisinin and its derivatives (ARTs) threatens the global efforts towards eliminating malaria. ARTs have been shown to cause ubiquitous cellular and genetic insults, which results in the activation of the unfolded protein response (UPR) pathways. The UPR restores protein homeostasis, which otherwise would be toxic to cellular survival. Here, we interrogated the role of DNA-damage inducible protein 1 (*Pf*Ddi1), a unique proteasome-interacting retropepsin in mediating the actions of the ARTs. We demonstrate that *Pf*Ddi1 is an active A_2_ family protease that hydrolyzes ubiquitinated proteasome substrates. Treatment of *P. falciparum* parasites with ARTs leads to the accumulation of ubiquitinated proteins in the parasites and blocks the destruction of ubiquitinated proteins by inhibiting the *Pf*Ddi1 protease activity. Besides, whereas the *Pf*Ddi1 is predominantly localized in the cytoplasm, exposure of the parasites to ARTs leads to DNA fragmentation and increased recruitment of the *Pf*Ddi1 into the nucleus. Furthermore, we show that Ddi1 knock-out *Saccharomyces* *cerevisiae* cells are more susceptible to ARTs and the *Pf*DdI1 protein robustly restores the corresponding functions in the knock-out cells. Together, these results show that ARTs act in multiple ways; by inducing DNA and protein damage and might be impairing the damage recovery by inhibiting the activity of *Pf*Ddi1, an essential ubiquitin-proteasome retropepsin.

## 1. Introduction

Artemisinin and its derivatives (ARTs) are components of mainstay drugs for the treatment of malaria caused by the *Plasmodium falciparum* parasite [1]. However, the emergence and spread of resistance towards the artemisinins poses an imminent danger towards the global efforts to eliminate malaria [2]. Historically, the spread of malaria drug resistance from South East (SE) Asia to India is a crucial “stepping stone” to the eventual introduction in Africa [3,4]. Regrettably, recent evidence has shown the presence of Artemisinin-resistant *P. falciparum* in India [5]. This situation does not only pose a grave danger to public health in these countries but also in sub-Saharan Africa, a continent most affected by malaria [1]. Whereas there is the most reliable evidence linking mutations in the Kelch domain protein (K13-propeller; PF3D7_1343700) with parasite tolerance to artemisinin [6], insufficient knowledge on the molecular mechanisms of artemisinin action hampers a definitive conclusion. Understanding the mechanisms of action and resistance of artemisinin, therefore, would not only provide a basis for identifying new targets but also be useful to the development of new alternative compounds that thwart and antagonize the emergence of resistance.

To date, the exact mechanism of action of the artemisinins remains debatable [7,8]. Artemisinin has been shown to directly interact with several *P. falciparum* proteins such as translationally controlled tumor protein homolog (*Pf*TCTP) and sarco/endoplasmic reticulum Ca^2+^-ATPase (*Pf*ATP6) [9]. Besides, heme-artemisinin adducts formed in the parasite have been shown to disrupt the formation of hemozoin [10]. Recent reports have demonstrated the promiscuous nature of artemisinin-mediated cellular damages [11,12,13,14,15]. For instance, besides the ubiquitous protein insults [16], Artemisinin has been attributed to DNA damage mediated by reactive oxygen species (ROS) [13]. Consequently, the damage would be expected to trigger stress response or unfolded protein response (UPR) pathways [17,18], such as the ubiquitin-proteasome system (UPS) [16]. The UPS degrades unfolded/damaged proteins that would otherwise be harmful to the cells. Interestingly, evidence has associated the K13-propeller protein with ubiquitination [12,19], and inhibitors of the UPS have been shown to enhance the action of artemisinin against the *P. falciparum* parasites [20,21,22]. Artemisinin inhibits the UPS and changes to this system mediate parasite tolerance to artemisinin pressure [16,20,23]. However, molecular data on the role of the UPS in mediating the action/resistance of the artemisinin in *P. falciparum* parasites remain scarce.

*Pf*Ddi1, an essential retropepsin (retroviral aspartyl protease) in the UPS [24,25], has been shown to compensate for proteasome dysfunction and its knock out leads to polyubiquitination of proteins in both yeast and *Toxoplasma gondii* cells [26,27,28]. It is feasible, therefore, to speculate that artemisinin might be compromising the activity of *Pf*Ddi1 in restoring protein homeostasis following the damage. Here, we identify the *Pf*Ddi1, commonly referred to as the proteasome shuttle protein, and investigate its role in mediating the actions of artemisinin. Binding and enzymatic assays demonstrate that *Pf*Ddi1 is an active proteasome reptropepsin that cleaves ubiquitinated substrates. We show that artemisinin enhances polyubiquitination of parasite proteins and inhibits the activity of *Pf*Ddi1 in digesting ubiquitinated proteins. In addition, the parasites’ exposure to artemisinin induces DNA fragmentation and increases recruitment of the *P*fDdi1 protein into the nucleus. Besides, using yeast complementation studies, we show that whereas *Sc*Ddi1 is dispensable in yeast, *Sc*Ddi1 deficient *S. cerevisiae* cells display more susceptibility to artemisinin pressure. The expression of *Pf*Ddi1 restores the functions in the corresponding Ddi1-knock out yeast cells. Our work thus gives insights into the role of the *Pf*Ddi1 in mediating the actions of ARTs and validates it as a vulnerable protein that could be the basis for the development of new chemotherapies against the *P. falciparum* malaria.

## 2. Results

### 2.1. PfDdI1 Is an Active A_2_ Family Protease That Hydrolyzes Polyubiquitin Substrates

**Figure 1:**
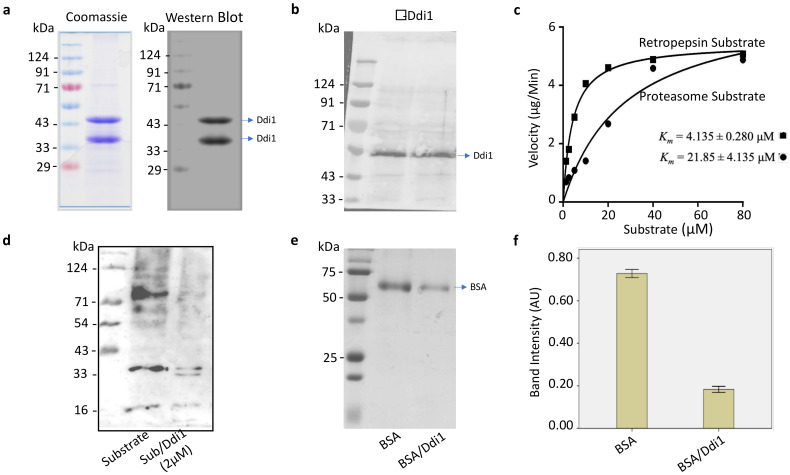
***Pf*Ddi1 hydrolyzes both peptide substrates and proteins.** (**a**) Coomassie-stained SDS-PAGE and Western blot analysis using α-His antibodies to detect the purified recombinant *Pf*Ddi1 (~44 kDa) and its processed fragment (~34 kDa). The soluble, recombinant protein was purified using the Ni-NTA resin (see also Figure S1a–c). (**b**) Mice or rabbit ant-Ddi1 antibodies detected a band of ~49 kDa from a parasite lysate. (**c**) Kinetics of substrate hydrolysis. Triplicate enzyme assays were carried out for 4 h at 37 °C. Both the DABCYL-Gaba-SQNYPIVQ-EDANS and Suc-LLVY-AMC cleavage signals were measured at an excitation wavelength of 355 nm and an emission wavelength of 460 nm. On the other hand, an excitation and emission wavelength of 340 and 425 nm, respectively, was used to monitor the hydrolysis of Bz-RGFFP- 4MβNA. The fluorescence signals were captured at 15-min intervals with the VICTOR Multilabel plate reader (VICTOR X3). The purified enzyme (*Pf*Ddi1) was more active on the retropepsin (DABCYL-Gaba-SQNYPIVQ-EDANS) substrate compared to the proteasome (Suc-LLVY-AMC) substrate. On the other hand, *Pf*Ddi1 did not hydrolyze the pepsin/cathepsin D (Bz-RGFFP-4MβNA; Figure S1d). (**d**) Western blot analysis showing the cleavage of polyubiquitin substrate (K^48^-linked substrate) by the *Pf*Ddi1 enzyme. The test assay (substrate/*Pf*Ddi1) or the control (substrate alone) were incubated at 37 °C for 2 h, and then resolved in a 12% SDS-PAGE. Cleavage of the polyubiquitin substrate was probed using rabbit anti-ubiquitin antibodies (U5379, Sigma Aldrich). (**e**) Coomassie stained SDS-PAGE showing degradation of BSA by the *Pf*Ddi1 enzyme. The test assay (BSA/*Pf*Ddi1) or the control (BSA alone) were incubated at 37 °C for 2 h, at pH 5.0, and then resolved in a 12% SDS-PAGE. (**f**) Quantification of the control and the degraded BSA band intensities (~66 kDa). There was no degradation of BSA at pH 7.0 (Supplementary Figure S3a). The units are arbitrary (AU) and the bars show the mean ± standard error for three independent reactions.

Whereas *P. falciparum* parasites express three proteasome interacting proteins (PIPs); *Pf*Ddi1, Rad23 and Dsk2, deletion of only *Pf*Ddi1 has been proven to be toxic to the cells, thus indispensable [24,25,29,30,31]. Compared to the other *Pf*PIPs, *Pf*Ddi1 harbors a unique retroviral-protease like (RVP) domain besides the conventional ubiquitin-like (UBL) domain. Despite being characterized in other organisms [26,27,32,33], Ddi1 remains poorly understood in *Plasmodium* spp. To functionally characterize the role of the *Pf*Ddi1, if any, we cloned and expressed a histidine-tagged full length *Pf*Ddi1 gene (PF3D7_1409300) in Rosetta (DE3) cells. The expressed recombinant *Pf*Ddi1 protein was analyzed by both Coomassie staining and Western blotting with α-His antibodies. The recombinant *Pf*Ddi1 protein was then purified under non-denaturing conditions, and it showed two discrete bands of ~44 kDa and ~34 kDa sizes on SDS PAGE, suggesting that the ~34 kDa band is probably a processed fragment of the *Pf*Ddi1 protein (Figure 1a and Supplementary Figure S1a–c). To know whether the ~34 kDa band is indeed a processed fragment of the intact *Pf*Ddi1 protein, we analyzed both bands by LC-MS/MS. The proteome analysis showed that the peptides identified in the LC-MS/MS analysis for each of the fragments corresponded to the *Pf*Ddi1 protein and interestingly, they both had the aspartic catalytic signature motif (DSG) (Supplementary Figure S2). The purified recombinant *Pf*Ddi1 protein was then used to raise antibodies in mice and rabbits. The specificity of the antibodies to detect native *Pf*Ddi1 was assessed by Western blot using trophozoite-rich *P. falciparum* blood stage parasite lysate. The mice or rabbit anti-*Pf*Ddi1 antibodies stained a band of the size expected for *Pf*Ddi1 in *P. falciparum (*Figure 1b). Since *Pf*Ddi1 possesses a retroviral-like protease (RVP) domain, we next assessed the pepsin/cathepsin D, retropepsin or proteasome activity of the purified recombinant *Pf*DdI1 protein using the Bz-RGFFP-MNA, DABCYL-Gaba-SQNYPIVQ-EDANS or Suc-LLVY-AMC substrates, respectively. The cleavage of the substrates and fluorescence signals were captured and used to measure the catalytic efficiency of the enzyme. Unlike the cathepsin D substrate, 2.0 μM of the enzyme hydrolyzed DABCYL-Gaba-SQNYPIVQ-EDANS or Suc-LLVY-AMC at pH 5.0. The enzyme was more active on the retropepsin substrate, with a catalytic efficiency of ~3.8 × 10^5^ M^−1^·s^−1^ (*K_m_* = 4.135 ± 0.280 μM), compared to the proteasome-specific substrate, with an efficiency of ~8.0 × 10^4^ M^−1^·s^−1^ (*K_m_* = 21.85 ± 4.135 μM) (Figure 1c and Supplementary Figure S1d). Due to its ability to hydrolyze the proteasome substrates, coupled with previous evidence that Ddi1 compensates for proteasome dysfunction [26], we hypothesized that the *Pf*Ddi1 might harbor the ability to degrade polyubiquitinated proteins/substrates. Polyubiquitination serves as a recognition signal for the proteasome. Our data showed that the incubation of K^48^-linked polyubiquitin substrate with 2.0 μM *Pf*Ddi1 enzyme led to significant cleavage of the substrate (Figure 1d). Together, these findings demonstrate that *Pf*Ddi1 is an active retroviral protease that hydrolyzes polyubiquitin/proteasome substrates.

### 2.2. PfDdi1 Enzyme Degrades BOVINE Serum Albumin, BSA

Having demonstrated the ability of the recombinant *Pf*Ddi1 protein to hydrolyze peptide substrates, we assessed the capacity of the enzyme to degrade macromolecules. Compared with the control (BSA alone), incubation of BSA with the *Pf*Ddi1 protein resulted in the degradation of BSA, at pH 5.0. SDS-PAGE analysis of the test assay showed a significantly reduced BSA band intensity (~66 kDa) (Figure 1e,f). On the other hand, the *Pf*Ddi1 could not hydrolyze BSA at pH 7.0 (Supplementary Figure S3a). Our data agree with previous findings that demonstrated a more favorable activity of *Leishmania major* Ddi1, at an acidic pH [34].

### 2.3. Artemisinin Increases Polyubiquitination in P. falciparum and Blocks the Activity of PfDdi1 in Degrading the Polyubiquitinated Substrates

**Figure 2:**
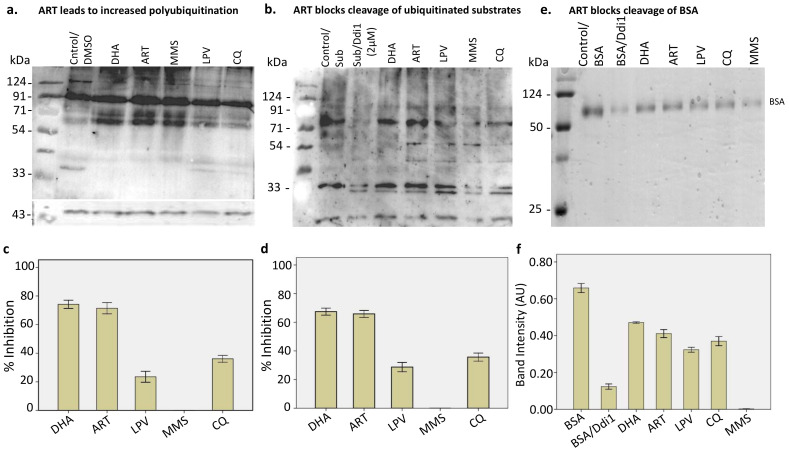
**Artemisinin exposure enhances protein ubiquitination and blocks the *Pf*Ddi1 activity.** (**a**) Western blot analysis showing increased ubiquitination in drug-treated *P. falciparum* parasites compared to the untreated control. Tightly synchronized mid-trophozoites were diluted to 5% parasitemia and then subjected to drug treatment (ART; 1 μM, DHA; 1 μM, MMS; 0.05% or LPV; 50 μM), for 4 h. DMSO was used as a vehicle treatment for all the assays and β-actin was used a loading control. The parasite lysates were resolved in a 10% SDS PAGE and probed with rabbit anti-ubiquitin antibodies. (**b**) ART and DHA (1 μM) blocked the cleavage of the polyubiquitin substrates. We incubated the polyubiquitinated substrate (K^48^-linked) with 2.0 μM of freshly purified recombinant *Pf*Ddi1 (as described previously) at pH 5.0. Triplicate assays and the control (substrate alone) were incubated at 37 °C for 2 h. The samples were resolved in a 12% SDS PAGE and probed with rabbit anti-ubiquitin antibodies (U5379, Sigma Aldrich). (**c,d**) Percentage inhibition of the *Pf*Ddi1 enzyme activity against the retropepsin (**c**) and proteasome (**d**) substrates, determined at 3 h. A total of 2.0 μM of the enzyme was preincubated with the drug compounds for 10 min before addition of the fluorescence substrates. The inhibition was expressed as a percentage of the control (see Supplementary Figure S3b,c) (**e**) Coomassie-stained SDS-PAGE showing inhibition of BSA degradation by *Pf*Ddi1. (**f**) Band intensity values of the inhibition of *Pf*Ddi1-catalyzed BSA degradation by the compounds. The intensity values are represented as arbitrary units (AU). The bars show means ± standard error for three independent reactions.

Artemisinin has been shown to cause widespread damage to parasite proteins [11,12,15]. The damage invokes the unfolded protein response pathways as a means of tidying up. Here, we assessed the impact of artemisinin on global protein ubiquitination as well as on the activities of the *Pf*Ddi1 enzyme. Exposure of trophozoite-rich 3D7 *P. falciparum* parasites to 1.0 μM of artemisinin (a physiologically relevant dose [35]) for 4 h resulted in the accumulation of polyubiquitinated proteins. Similarly, Dihydroartemisinin (DHA; 1.0 μM, the most active artemisinin metabolite) and Methyl methanesulfonate (MMS; 0.05%) led to enhanced polyubiquitination, but not Lopinavir (50 μM) (Figure 2a). MMS induces DNA strand breaks by alkylating DNA bases [14] and has been associated with the production of ROS in cells [36]. The rapid protein polyubiquitination under artemisinin pressure invoked thoughts about its potential inhibition ability against the *Pf*Ddi1 enzyme activities. As expected, artemisinin and its derivative, DHA, significantly inhibited the ability of *Pf*Ddi1 to degrade the polyubiquitinated substrate. Besides, artemisinin significantly inhibited the activity of *Pf*Ddi1 with both the retropepsin—(71.4%) and proteasome—(65.9%) specific substrates, as well as with BSA (Figure 2b–f and Supplementary Figure S3b,c). These data are in sync with previous observations which showed that artemisinin directly binds and inhibits the activity of *Pf*ATP6 in intact cells and the activity of heterologously expressed *Pf*ATP6 enzyme as well as alkylation of the *Pf*TCTP [9]. Suprisingly, Lopinavir (50 μM), a known HIV protease inhibitor that was expected to significantly block the activity of *Pf*Ddi1, produced about 23% inhibition. In comparison, whereas MMS (0.05%) treatment led to increased polyubiquitination, it seemed to enhance the activity of *Pf*Ddi1 protein against all the substrates (Figure 2b–f). These data demonstrate the dual mechanisms of action of the artemisinins; by promoting protein damage in the parasite and potentially blocking tidying up by inhibiting the activities of *Pf*Ddi1 enzyme.

### 2.4. Artemisinin Treatment Leads to Increased Recruitment of PfDdi1 into the Nucleus following DNA Damage

**Figure 3:**
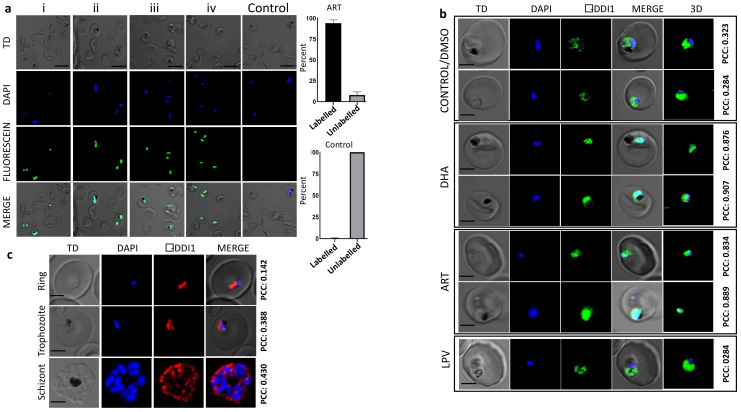
**Causal relation between artemisinin-specific DNA fragmentation and *Pf*Ddi1 subcellular localization.** (**a**) Representative IFA images showing that artemisinin induces DNA fragmentation (i–iv) in *P. falciparum* parasites. The parasites were subjected to drug pressure for 2 h and then the damage was assayed using TdT-mediated dUTP nick end labelling. The percentage (average) of cells with DNA breaks (TUNEL-positive cells) was estimated by counting the breaks (green fluorescence) or the TUNEL-negative nuclei in several fields (*n* = 200). Fragmentation was observed in more than 90% of the infected RBCs. (**b**) Increased recruitment of *Pf*Ddi1 into the nucleus following ART or DHA pressure compared with control (DMSO) or LPV. The IFA staining was performed using mice anti-*Pf*Ddi1 antibodies and then underwent 3D reconstruction in IMARIS software. (**c**) IFA staining of *P. falciparum* blood stage parasites with rabbit anti-*Pf*Ddi1 antibody showing constant expression and localization of Ddi1 in the cytoplasm. Pearson correlation coefficient (PCC) values were calculated from the fluorescence intensities to indicate the degree of spatial co-localization between the *Pf*Ddi1 and DAPI (a nucleus specific stain). The individual stains, merged images, and bright field are shown. Scale bars: 5 μm. The experiments were performed on two to four independent occasions with technical duplicates. See also Supplementary Figure S4.

Artemisinin has been shown to induce DNA damage in malaria parasites as demonstrated by comet assays [13]. However, data on the nature of the DNA damage remain elusive. Using an in situ DNA fragmentation (TUNEL) assay, we observed DNA fragmentation (green fluorescence; direct TdT-mediated dUTP nick end labeling) in more than 90% of the *P. falciparum* parasites following a two-hour exposure to artemisinin (Figure 3a). The percentage (average) of cells with DNA breaks (TUNEL-positive nuclei) was estimated by counting the breaks (green fluorescence) against the total cells in several random fields (*n* = 200). To gain insights into the possible molecular events accompanying the artemisinin-specific DNA fragmentation, we employed immunofluorescence assays (IFA), using anti-*Pf*Ddi1 antibodies, to evaluate the expression profile of the *Pf*Ddi1 protein, under drug pressure. Previously, Ddi1 was shown to repair DNA-protein crosslinks (DPCs) in yeast cells [32]. Our data showed that, whereas *Pf*Ddi1 is predominantly expressed in the cytoplasm, artemisinin and DHA treatment led to increased recruitment of the protein into the *P. falciparum* nucleus as demonstrated by the Pearson correlation coefficients (PCC) (Figure 3b,c and Supplementary Figure S4). Thus, whereas we have not shown the exact DNA repair mechanism, the shift in expression is likely to be a causal relation between the DNA fragmentation and *Pf*Ddi1 repair strategies. To confirm that the phenotypes observed in IFA assays are not due to formation of pyknotic parasite cells, we performed a time-point microscopic analysis of parasite morphologies on thin smears. The analysis demonstrated that, although the parasites under artemisinin pressure demonstrated growth retardation compared to the control cells (no drug), significant pyknotic forms were seen after 8 h of treatment (Supplementary Figure S5).

### 2.5. Artemisinin Binds and Interacts with the Highly Conserved Aspartic Protease Motif “DSG” in PfDdi1 Protein

**Figure 4:**
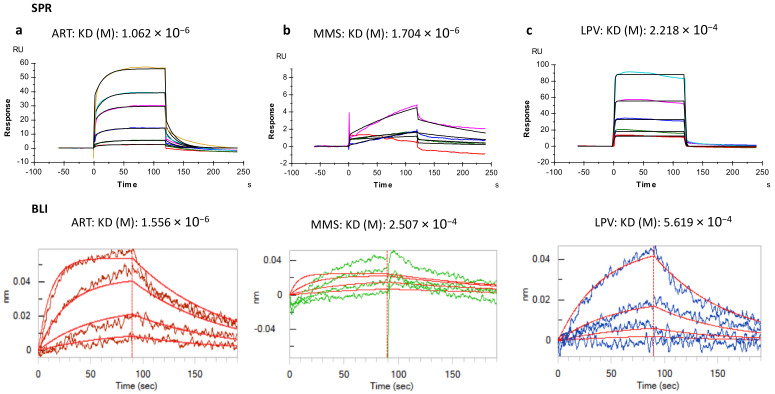
**Artemisinin binds to the recombinant *Pf*Ddi1 protein: SPR and BLI assays**. Over 8500 response units (RU) or up to a maximum of 0.8 nm shift of the recombinant *Pf*DdI1 was immobilized via the amine coupling chemistry (CM5 chip) or streptavidin-biotin capture (Octet biosensors), respectively. Binding of artemisinin (ART; (**a**)) and Methylmethanesulfonate (MMS; (**b**)) showed high affinity interactions with *Pf*Ddi1, compared to Lopinavir (LPV; (**c**)), as shown by the K_D_ values. In addition, none of the compounds showed interaction with the Heme detoxification protein (HDP; see Figure S6). Three independent SPR or BLI experiments were performed and representative binding sensorgrams are presented. Both the real time binding curves and the global 1:1 fits are shown. The binding kinetics data were analyzed using the Biacore T200 evaluation software v3.1 or the Octet Software v10.0.

Having established the effect of artemisinin on the *Pf*Ddi1 protease activity, we carried out surface plasmon resonance- (SPR) and Bio-Layer Interferometry- (BLI) based binding assays, as well as computational analysis to delineate the interaction between *Pf*Ddi1 and artemisinin. Briefly, over 8500 response units (RU) or up to a maximum of 0.8 nm shift of the recombinant *Pf*DdI1 was immobilized via the amine coupling chemistry (CM5 chip) or streptavidin-biotin capture (Octet biosensors), respectively. Depending on the buffer in which the compounds were dissolved, we used either HBS-EP or DMSO as running buffer. Both artemisinin and MMS showed high affinity interactions with *Pf*Ddi1 in both binding assays with k_D_ values of 1.062 × 10^−6^/1.556 × 10^−6^, and 1.704 × 10^−6^/2.507 × 10^−4^, respectively, while lopinavir showed lower binding affinities with a k_D_ value 2.218 × 10^−4^/5.619 × 10^−4^, in SPR and BLI assays (Figure 4a–c). To test the specificity of artemisinin, MMS or LPV in binding the *Pf*Ddi1 protein, we assessed the binding of these compounds to heme detoxification protein (HDP). The data showed that none of the compounds interacted with HDP (Supplementary Figure S6). On the other hand, PFAM and INTERPROSCAN search revealed the presence of two conserved domains in the *Pf*Ddi1 sequence: N-terminal Ubiquitin-like domain (4-74aa) and a retroviral-like protease domain (RVP; 222-345aa) (Supplementary Figures S1a and S7). Further, to understand conservation among different species, we performed multiple sequence alignment of Ddi1 sequences from *P. falciparum*, *L. major*, yeast and human. The alignment analysis showed that *Pf*Ddi1 had 95% query coverage and ~29% identity with human Ddi1 (hDdi1). In addition, all the aligned Ddi1 protein sequences showed higher conservation in the central RVP domain region as compared to the N- or C-terminal regions, with the presence of superimposed highly conserved aspartyl protease signature motif “D(S/T)G” (Supplementary Figure S7).

**Figure 5:**
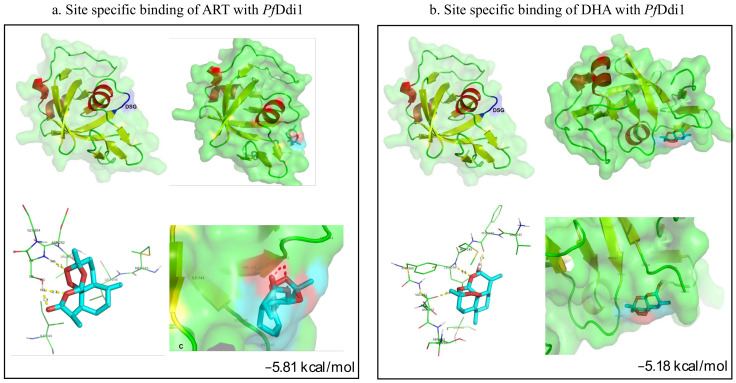
**Site-specific molecular docking using predicted *Pf*Ddi1 structure as a receptor and an artemisinin** (**a**) and a dihydroartemisinin molecule (**b**) as ligands. The *Pf*Ddi1 protein binds with both artemisinin (−5.81 kcal/mol) and dihydroartemisinin (−5.18 kcal/mol) in the highly conserved aspartic protease motif “DSG”. The *Pf*Ddi1 residues present within 4 Å of artemisinin and dihydroartemisinin and are involved in direct interaction with the compounds. Homology-based 3D model of the *Pf*Ddi1 RVP domain as generated by SWISSMODEL. The conserved Aspartic protease motif DSG is present in the coil region. Red—helix, green—coil, yellow—sheet and blue—conserved active motif.

As no crystal structure for *Pf*Ddi1 protein is available so far, we generated a homology-based 3D model for *Pf*Ddi1. All attempts to generate complete stable 3D structure for *Pf*Ddi1 (382aa) were futile. We then generated a partial 3D model for the *Pf*Ddi1 RVP domain (243-366aa) using 4RGH, a human Ddi1 Homolog 2 protein, having 37% query coverage and 48.61% identity with the *Pf*Ddi1, as a template (Figure 5). The Ramachandran plot for the predicted model showed no residues in the disallowed region, confirming the good quality of the model. To assess whether an artemisinin molecule or its derivative, dihydroartemisinin, binds to the protease domain region, we performed in silico docking using AutoDocktools. Here, site-specific docking was performed using the predicted *Pf*Ddi1 as a receptor and an artemisinin or dihydroartemisinin molecule as a ligand. Grid box was generated using nitrogen of Asp262 as the center (grid points xyz coordinates as 40, 40, 40 and spacing of 0.4 Å), and the other default parameters were used for the screening. The docking analysis revealed that the *Pf*Ddi1 protein binds with both artemisinin and dihydroartemisinin in the active catalytic protease signature motif (DSG), with all the three active-site residues of the aspartyl proteases present within 4 Å of both compounds (Figure 5). The free binding energy for the reaction between the artemisinin molecule and *Pf*Ddi1 was −5.81 kcal/mol (Figure 5a). Interestingly, the binding analysis between the dihydroartemisinin molecule and *Pf*Ddi1 yielded a free binding energy of −5.18 kcal/mol, which was comparable with that shown with artemisinin (Figure 5b). In comparison, site-specific interaction with MMS or LPV yielded a free binding energy of −2.67 or −1.43 kcal/mol, respectively, demonstrating weak binding affinity (Supplementary Figure S6d). Taken together, the SPR, BLI binding assays along with docking results suggest interaction between the *Pf*Ddi1 protein and the artemisinins, and possible inhibition of the *Pf*Ddi1 protease activity by artemisinin.

### 2.6. PfDdi1 Restores the Protein Secretion Phenotype in Yeast Cells

**Figure 6:**
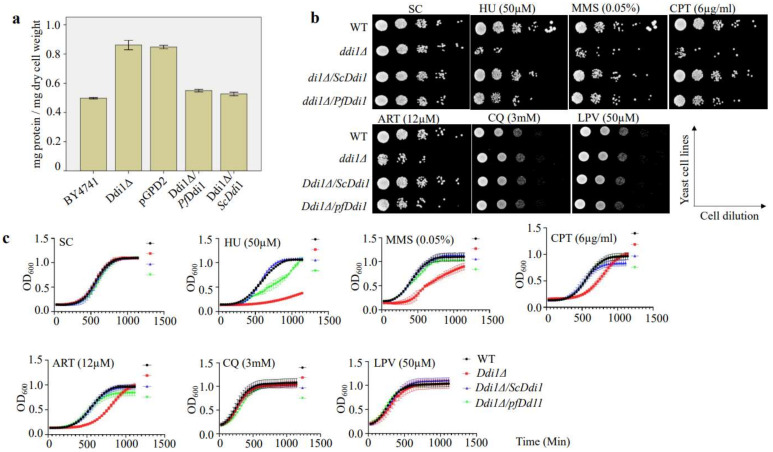
**Ddi1 deficient yeast cells are more susceptible to artemisinin pressure.** (**a**) We singly disrupted the *Sc*Ddi1 gene, by homologous recombination, and assayed whether the *Pf*Ddi1 ortholog could complement the phenotypes in the knockout yeast cells. Ddi1 deficient yeast cells secret high levels of proteins into the media and *Pf*Ddi1 reverts the protein secretion phenotype to wild type, as did the *Sc*Ddi1 construct. The protein content in the supernatant was estimated using the Pierce BCA Protein Assay Kit and the protein concentration expressed as milligrams of protein secreted per milligram of dry cell weight. Three independent experiments with triplicate assays were conducted, and the bars represent means ± standard error. Spot test images (**b**) and growth curve assays (**c**) showing the effect of the compounds on the different yeast lines. We incubated equal amounts of yeast cells with different drugs and DNA damaging agents at 30 °C for 48 h and then measured sensitivity using both OD (growth curves) and spotting tests. Whereas deletion of Ddi1 did not affect the growth fitness of the yeast cells, Ddi1 deficient cells were more susceptible to artemisinin.

To know whether the *Pf*Ddi1 is a true orthologue of yeast Ddi1, we performed complementation studies in *S. cerevisiae* yeast cells. We singly disrupted the *Sc*Ddi1 gene, by homologous recombination, and assayed whether the *Pf*Ddi1 ortholog could complement the phenotypes in the knockout yeast cells. Cells bearing a Sc*Ddi1* gene disruption were seen to grow normally. However, as has been shown previously [33], our data showed that *Ddi1*Δ yeast cells secreted significantly higher protein levels into the media (Figure 6a). On average, *Ddi1*Δ yeast cells secreted more than ~30% of proteins into the media, compared with the wild type strain. To test whether *Pf*DdI1 restores the wild-type proteins secretion phenotype, we cloned a gene encoding the full-length *Pf*Ddi1 into a yeast expression vector pGPD2. The ligated construct was transformed into the *DdI1*Δ yeast cells. The *Pf*DdI1 construct was able to revert the protein secretion phenotype to WT level, i.e., the level of protein secretion decreased in comparison to the knock-out strain (Figure 6a).

### 2.7. Ddi1 Deficient Yeast Cells Are More Susceptible to Artemisinin Pressure

It has been previously shown that mutations in some DNA repair genes confer resistance to artemisinin [37]. Moreover, DNA damaging agents have been shown to perturb and induce transcriptional changes in 21% of the *P. falciparum* genome [14]. These changes involve up-regulation of the genes of the DNA repair machinery. Similarly, yeast studies demonstrated that DNA damaging agents trigger differential expression in one third of the entire *S. cerevisiae’s* gene pool [38]. We reasoned that since the proteasome system is central to the repair or disposal of damaged cellular components, the yeast cells lacking the Ddi1 might be more susceptible to DNA damaging agents. We, therefore, incubated equal amounts of yeast cells with different drugs and DNA Damaging agents such as artemisinin, chloroquine, lopinavir, hydroxyurea, MMS, and camptothecin and measured sensitivity using both OD (growth curves) and spotting tests. Our results demonstrated that Ddi1Δ yeast cells were more susceptible to artemisinin (12 μM). These Ddi1Δ cells were also hypersensitive to DNA damage drugs; Hydroxyurea, MMS, and Camptothecin (Figure 6b,c). Together, these results augment our observations in *P. falciparum* and demonstrate that *Pf*Ddi1 reduces the sensitivity of the cells to artemisinin insults, and that artemisinin might be working like the known DNA damaging agents.

## 3. Discussion

The spectrum of drugs to which the human malaria parasite, *Plasmodium falciparum* has not evolved tolerance is rapidly diminishing. Reports on decreased sensitivity of the parasites towards the recommended first-line treatment for *P. falciparum* malaria, artemisinin, threatens the global efforts to combat the disease. To circumvent the resistance, improve the efficacy or generate new drugs, it is critical to understand the mechanisms of action/resistance of the artemisinin. Since it has been shown that artemisinin causes indiscriminate damage to parasite cellular proteins [11,12,13,14], recruitment of the parasite protein repair machinery might be pivotal in assuring parasite growth fitness. Besides, artemisinin compromises the functions of the parasite proteasome and synergizes with proteasome inhibitors in the killing of artemisinin resistant parasites [16,22]. Here, we investigated the effect of artemisinin on the parasite proteasome machinery. Expression and activity analysis of *Pf*Ddi1, a proteasome shuttle protein with an unusual RVP domain, showed that *Pf*Ddi1 is an active A_2_ aspartyl protease that hydrolyzes proteasome substrates, including polyubiquitin proteins. However, the enzyme could not catalyze the hydrolysis of Bz-RGFFP-MNA, a cathepsin D substrate. This activity is in line with earlier reports that showed *L. major* Ddi1 as an active aspartyl proteinase [34], as well as *Sc*Ddi1 as a ubiquitin-dependent protease that acts on polyubiquitinated substrates [27]. The ability of *Pf*Ddi1 to cleave polyubiquitinated substrates suggests that the *Pf*Ddi1 enzyme might not only be a shuttle protein but could inherently degrade damaged proteins. Thus, the *Pf*Ddi1 protein might be acting synergistically with the proteasome machinery to degrade the ubiquitinated proteins. Indeed, previous reports have demonstrated that Ddi1 compensates for proteasome dysfunction in *Caenorhabditis elegans* [26]. In addition, deletion of Ddi1 (ΔDdi1) in *T. gondii* results in the accumulation of ubiquitinated proteins, a phenomenon enhanced by double deletion (ΔDdi1 and ΔRad23) [28]. Therefore, the essentiality of the *Pf*Ddi1 advocates its multiple roles in parasite life cycle and negates any redundancy in its functions. On the other hand, in silico data reveal that unlike most of the Ddi1 analogs, *Pf*Ddi1 lacks the UBA domain, thus suggesting that the UBA domain does not contribute to the protease activity of the Ddi1 protein. These results are consistent with the observation made earlier for *Sc*Ddi1, which showed that the deletion of UBA domain had no effect on the activity of *Sc*Ddi1 [27].

To further show that *Pf*Ddi1 is a functional homolog of the *Sc*Ddi1, which is one of the best characterized Ddi1 proteins, complementation studies were carried out in *S. cerevisiae* cells. Functional expression of the *Pf*Ddi1 in *S. cerevisiae* cells showed its ability to restore disrupted phenotypes. We show that, unlike in *P. falciparum* where functional disruption of Ddi1 gene is deleterious, *Sc*Ddi1 gene is not refractory to deletion in yeast cells. However, as previously reported [33], deletion of *Sc*Ddi1 increased secretion of proteins to the growth media. Interestingly, despite the differences in the domain structure, the *Pf*Ddi1 gene robustly complemented the yeast secretion phenotype. This observation thus suggested that the C-terminal UBA domain lacks crucial sequences associated with the suppression of protein secretion.

Since artemisinin has been shown to promiscuously target parasite proteins and induce DNA damage as demonstrated by the Comet assay [13], we sought to define whether it also inhibits the activity of *Pf*Ddi1. Besides, artemisinin has been shown to inhibit *Pf*ATP6 in intact parasites just as it quashes the ATPase activity of heterologously expressed *Pf*ATP6 enzyme [9]. We first demonstrated that, indeed, artemisinin causes protein damage which leads to piling up of polyubiquitinated proteins. These data agree with previous data which showed that the artemisinins induce polyubiquitination in the malaria parasites [16,20]. In parallel, we demonstrated that artemisinin causes DNA damage by directly inducing DNA fragmentation in the *P. falciparum* parasites. Interestingly, the exposure of the parasites to genotoxic artemisinin insults causes increased recruitment of *Pf*Ddi1 into the nucleus. These results suggest that *Pf*Ddi11 might be involved in the regulation of DNA damage response to artemisinin. Indeed, previous reports have implicated Ddi1 in the repair of DNA-protein crosslinks [32]. The exact mechanism adopted by the *Pf*Ddi1 in the DNA damage repair in the parasites remains of utmost interest. Enzyme inhibition assays showed that artemisinin blocked 71.4% or 65.9% of the activity of *Pf*Ddi1 against the retropepsin or proteasome substrates, respectively. Besides, artemisinin significantly inhibited the degradation of polyubiquitinated substrates, a finding that unequivocally fortifies the inhibitory effect of artemisinin on the *Pf*Ddi1 activities. These findings are in sync with the previous data which showed that the artemisinins could block the Ca^2+^- dependent ATPase activity of PfATP6, expressed in a heterologous system [9]. On the other hand, lopinavir (50 μM), a known HIV protease inhibitor, could only yield marginal inhibition (~23.5%) of the activity of *Pf*Ddi1, a retroviral aspartyl protease. In addition, interaction sensograms from binding assays and in silico modeling and docking studies showed high affinity binding between the artemisinin molecule and *Pf*Ddi1. Similarly, DHA, the most potent artemisinin derivative, yielded a binding affinity towards *Pf*Ddi1 which was comparable with that of artemisinin. These data demonstrate that both artemisinin and its derivative (DHA) could directly bind and inhibit the activity of the *Pf*Ddi1 enzyme.

To provide additional evidence on the role of Ddi1 in the mediation of artemisinin activities, we studied the growth fitness of Ddi1 deficient *S. cerevisiae* (*Sc*DdI1Δ) cells. This transgenic line showed differential susceptibilities to an array of DNA damage compounds as well as to artemisisnin, the mainstay anti-malarial drug. *Sc*DdI1Δ cells were more susceptible to artemisinin pressure, compared to the wild type cells. Therefore, the observed increased susceptibility might be because of the *Sc*DdI1Δ cells’ inefficiency to invoke DNA and protein repair upon artemisinin-induced damage. In fact, artemisinin has been shown to increase the generation of free radicals that chokes the yeast cell homeostasis [39]. Besides, artemisinin has been shown to elicit DNA damaging effect comparable to MMS, an alkylating agent [14]. Restoration of the WT growth fitness by the *Pf*Ddi1 infers that the UBA domain plays an insignificant role in responding to artemisinin genotoxic insults.

**Figure 7:**
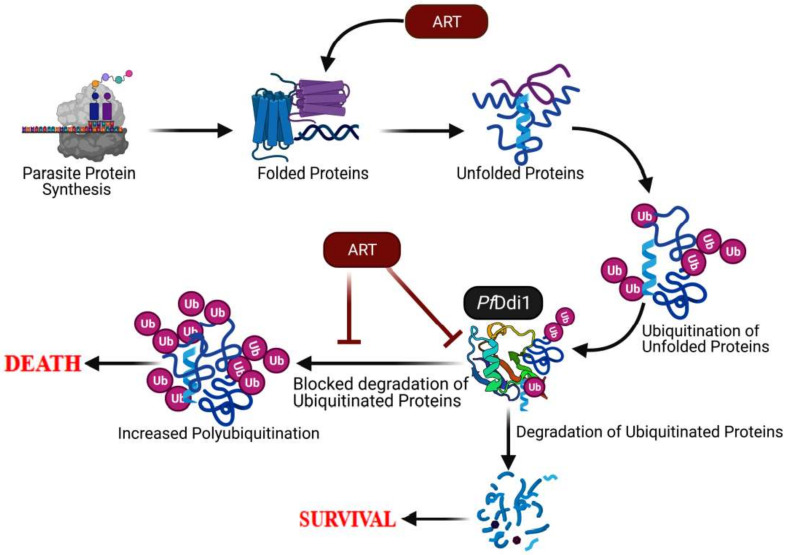
**Model demonstrating ART-induced killing of *Plasmodium* parasites.** ART has been shown to target several parasite proteins and processes. Here, we focus on the role of the *Pf*Ddi1 in mediating the actions of ART. The ART’s ubiquitous damage of parasite proteins leads to the need for tidying up via the *Pf*Ddi1 or proteasome machinery. Besides causing the protein damage, artemisinin binds to *Pf*Ddi1 and blocks the degradation of the damaged proteins. Besides, ART might be preventing the trafficking of the damaged proteins to the proteasome for degradation.

These results thus enhance the evidence on the mode of action of artemisinin that has been earlier shown to kill the parasites via a two-step mechanism; causing ubiquitous protein damage and compromising parasite proteasome functions [16]. Therefore, based on our data coupled with the previous observations, we propose that artemisinin might be exerting its pressure on the parasite by compromising the *Pf*Ddi1 protein, an important player in the parasite protein homeostasis. The compromised *Pf*Ddi1 does not only lose its ability to degrade the damaged proteins but also curtail its shuttling capacity, thus leading to accumulation of the damaged proteins and eventual death of the malaria parasite (Figure 7).

## 4. Materials and Methods

### 4.1. Ethics Statement

All protocols were conducted in accordance with prior approvals obtained from the International Centre for Genetic Engineering and Biotechnology (ICGEB)’s Scientific Ethical Review Unit and the Institutional Animal Ethics Committee (IAEC; ICGEB/IAEC/02042019/MB-7).

### 4.2. Cloning, Expression, and Purification of Recombinant PfDdi1

The *Pf*DdI1 gene (PF3D7_1409300) was amplified from genomic DNA using specific primers; DdI1 forward 5′-GCGGATCCATGGATATGGTTTTTATTACAATATCAGACG-3′, reverse 5′-GCGTCGACCTCGAGTAAATCATTGTTTGCATCAATG-3′. The PCR product was first cloned into pJET vector (Thermo Scientific, Massachusetts, MA, USA) and then sub-cloned into pET-28b expression vector using NcoI and XhoI restriction sites (Thermo Scientific). The pET-28b clone was expressed in Rosetta (DE3) *Escherichia coli* cells (Invitrogen, Waltham, MA, USA). The cells were grown to mid log phase, and then induced with isopropyl-1-thio-β-D galactopyranoside (IPTG, 1 mM) for 14 h at 16 °C. The bacterial culture was harvested by centrifugation at 4000× *g* for 20 min. The cell pellet was re-suspended in lysis buffer (50 mM Tris·HCl at pH 8.0, 200 mM NaCl, 1.0% Triton X-100 and 1.0% PMSF) and then sonicated. The supernatant was collected by centrifugation at 9000 rpm for 50 min, at 4 °C. The soluble recombinant protein was purified using the Nickel-Nitrilotriacetic acid (Ni-NTA^+^; Qiagen, Hilden, Germany) resin. Briefly, the protein was allowed to bind in 20 mM imidazole-containing binding buffer (50 mM Tris:HCl at pH 8.0 and 200 mM NaCl) for 3 h at 4 °C. The resin with bound protein was washed in 30 mM imidazole-containing binding buffer and then the bound protein was eluted in varying concentrations of the imidazole (50, 75, 100, 150, 200, 300 and 500 mM) in 50 mM Tris:HCl at pH 8.0 and 200 mM NaCl. The purified fractions were checked on SDS-PAGE and Western blot analysis using α-His antibodies. All the pure fractions were pooled and dialyzed in the Tris-Nacl buffer (50 mM Tris-Hcl pH 8, 200 mM Nacl), and then concentrated.

### 4.3. Generation of Antibodies against PfDdi1

All animal protocols were conducted in accordance with prior approvals obtained from the International Centre for Genetic Engineering and Biotechnology (ICGEB)’s scientific review committee and the institutional animal ethics committee (ICGEB/IAEC/02042019/MB-7).

We used BALB/c inbred mice and female NZW rabbits to raise anti-bodies against the recombinant *Pf*Ddi1. The mice were immunized with 20 μg of the protein while the rabbits were immunized with 200 μg protein in the presence of complete/incomplete Freund’s adjuvant, using the i.p. and s.c. modes of injection, respectively. After the third bleed, the antibody titers were quantified by ELISA. The specificity of the raised antibodies was analyzed on the recombinant *Pf*Ddi1 protein and the *P. falciparum* parasite lysate.

### 4.4. PfDdi1 Enzymatic Assays

The aspartyl proteinase activity of the purified recombinant *Pf*DdI1 was probed against three substrates; Bz-RGFFP-4MβNA, DABCYL-Gaba-SQNYPIVQ-EDANS or Suc-LLVY-AMC (Bachem, Bubendorf, Switzerland), following the protocol described earlier [33]. Briefly, 2.0 μM of the recombinant *Pf*DdI1 was incubated with decreasing concentration (80–1.25 μM) of each of the substrates in 100 mM sodium acetate buffer, at pH 5.0. Triplicate assays were carried out in a total volume of 200 μL in 96 well opaque plates, for 4 h at 37 °C. Assays with Heme Detoxification Protein (*Pf*HDP) were used as the control. Both the DABCYL-Gaba-SQNYPIVQ-EDANS and Suc-LLVY-AMC cleavage signals were measured at an excitation wavelength of 355 nm and an emission wavelength of 460 nm. On the other hand, an excitation and emission wavelength of 340 and 425 nm, respectively, was used to monitor the hydrolysis of Bz-RGFFP- 4MβNA. The fluorescence signals were captured at 15-min intervals with the VICTOR X3 Multilabel plate reader (PerkinElmer, Waltham, MA, USA). Due to the intrinsic reduction in fluorescence associated with fluorescence resonance energy transfer (FRET)-based cleavage assays, fluorescence from varied concentrations of free Edans (from 0.625 to 40 μM) in the assay buffer was used to generate a standard calibration curve and for correction of the inner filter effect [40,41]. The obtained relative fluorescence units were converted into velocity {μg (cleaved substrate)/s} and then used to derive the kinetic and catalytic constants in GraphPad Prism v6.0. The enzyme’s overall ability to cleave the substrate was represented as k_cat_/K_M_ (M^−1^ s^−1^).

### 4.5. Proteolytic Assays on Polyubiquitin Substrates and Macromolecules

We incubated 20 μg of polyubiquitin substrate (K^48^-linked) or 0.25 mg/mL of bovine serum albumin with 2.0 μM of freshly purified recombinant *Pf*Ddi1 in the 50 mM sodium acetate buffer (as described previously), pH 5.0, in a final volume of 100 μL. Triplicate assays and the control (substrate alone) mixture were incubated at 37 °C for 2 h. The mixtures were centrifuged and then resolved in a 12% SDS-PAGE. Cleavage of the polyubiquitin substrate was probed using rabbit anti-ubiquitin antibodies (U5379, Sigma Aldrich, St. Louis, MO, USA) and then detected by enhanced chemiluminescence (ECL) using the Bio-Rad ChemiDocTM MP imaging. On the other hand, cleavage of the BSA substrate was stained by Coomassie brilliant blue. The arbitrary band intensity values were presented as means ± standard error (SE).

### 4.6. In Vitro Culture of Plasmodium falciparum and Drug Treatment

*P. falciparum* parasites (3D7 strain) were cultured and maintained in purified human red blood cells at 4% hematocrit, in RPMI 1640 medium (Gibco; Thermo Scientific, Waltham, MA, USA) supplemented with 0.25% Albumax I (Gibco), 2 g/L Sodium bicarbonate (Sigma), 0.1 mM hypoxanthine (Sigma), and gentamicin (Gibco). Parasite cultures were kept at 37 °C with 5% CO_2_, 3% O_2_, and 92% N_2_. The parasites (ring stage; 2–4 hpi) were tightly synchronized with 5% (*v*/*v*) d-sorbitol (Sigma) and then monitored by Giemsa staining of methanol-fixed blood smears. Tightly synchronized mid-trophozoites were diluted to 5% parasitaemia and then subjected to the drug treatment (artemisinin; 1 μM, DHA; 1 μM, MMS; 0.05% or LPV; 50 μM), for 4 h. DMSO was used as a vehicle treatment for all the assays. The parasite cell pellets were washed with ice-cold PBS and then lysed with 0.15% (*w*/*v*) saponin and radioimmunoprecipitation assay buffer (RIPA buffer) as described previously. The protein content was normalized with BCA assay and then resolved by a 10% SDS PAGE. The gel was transferred to a nitrocellulose membrane blocked with 5% (*w*/*v*) skim milk for 1 h at room temperature and probed with primary rabbit anti-ubiquitin antibody (1:100) overnight at 4 °C, followed by HRP-conjugated secondary antibody for 1 h at room temperature. The blots were processed by ECL reagents and then detected using the Bio-Rad ChemiDocTM MP imaging (Bio-Rad, Hercules, CA, USA).

### 4.7. Enzyme Inhibition Assays

For the enzyme inhibition assays, we preincubated 2.0 μM of the enzyme with drug compounds {artemisinin (1 μM), DHA (1 μM) MMS (0.05%) or LPV (50 μM), in sodium acetate buffer, pH 5.0 for 10 min, at ~24 °C. We then added 10 μM of the fluorescence substrates or 20 μg of polyubiquitin protein and the inhibition experiments were carried out at 37 °C. The fluorescence signals and the protein degradation were processed as earlier described. The experiments were carried out in triplicates and fluorescence inhibition was expressed as a percentage of the control.

### 4.8. Protein-Drug Interaction Assays (Optical Methods; SPR and BLI)

All the SPR or BLI experiments were performed using a T200 instrument (Biacore, Uppsala, Sweden) or the Bio-Layer Interferometry (BLI) Octet RED96e platform (FortéBio, Fremont, CA, USA). Freshly prepared HEPES-buffered saline (*HBS)-*EP (0.01 M HEPES; pH 7.4, 0.15 M NaCl, 0.003 M EDTA, 0.05% vol/vol P20 surfactant) or DMSO was used as a running buffer for the experiments. For the SPR interaction assay, over 8500 response units (RU) of the recombinant *Pf*DdI1 in sodium acetate buffer (pH 4.5) were immobilized on a SPR CM5 sensor via amine coupling [42]. A blank flow cell was used for reference corrections. Heme detoxification protein (HDP) was also immobilized on the CM5 sensor and used as the control protein. For the BLI interaction assay, biotinylated*Pf*Ddi1, diluted to a concentration of 25 μg/mL in kinetics buffer (HBS-EP with 0.1 mg/mL BSA) was immobilized on streptavidin-coated (SA) biosensors (FortéBio). The ligand was immobilized up to a maximum of 0.8 nm shift. Reference biosensors loaded with the ligand but dipped into wells containing only the buffer were run in parallel to control for possible drifts and the establishment of a baseline. Serial two-fold dilutions of the compounds, artemisinin (1 μM), lopinavir (50 μM) or MMS (0.05%), diluted in the running buffer, were used and the kinetics performed at 25 °C. In the SPR experiments, a total of 0.2 mL of the sample was injected while in BLI, each biosensor was stirred in 0.2 mL of the sample at 1000 rpm. The kinetics data were analyzed using the Biacore T200 evaluation software v3.1 or the Octet Software v10.0. The affinity between the immobilized protein and the compounds was expressed as dissociation constant (K_D_).

### 4.9. PfDdi1 3D Model Generation and In Silico Docking

PlasmoDB (https://plasmodb.org/plasmo/; accessed on 5 April 2021) and UniProt (https://www.uniprot.org/; accessed on 5 April 2021) were used to retrieve sequences for Ddi1 proteins [43]. Artemisinin chemical structure was retrieved from PubChem database (https://pubchem.ncbi.nlm.nih.gov/ accessed on 6 April 2021). To identify conserved domains in the *Pf*Ddi1 protein, we performed PFAM and INTERPROSCAN search [44,45]. We then used CLUSTAL-Omega version 1.2.4 to perform multiple sequence alignment [46]. SWISS-model was used to generate a three-dimensional (3D) model for *Pf*Ddi1 [47], and then Rampage tool was used for quality check analysis for the predicted model [48]. AutoDock tools were used to perform docking analysis [49] and the generated images of the molecular models were visualized using PyMol (https://pymol.org/2/; accessed on 6 April 2021).

### 4.10. Immunofluorescence Assay

Immunofluorescence assay (IFA) was performed with *Plasmodium* parasite cells in suspension. Briefly, parasite pellet was washed in 1 × PBS and fixed in 4% *v/v* formaldehyde supplemented with 0.0075% *v/v* glutaraldehyde in PBS for 30 min at RT. The cells were then permeabilized in 0.1% Triton X-100 in PBS for 20 min and then washed in 1 × PBS. We then blocked with 5% BSA for 1 h at room temperature, and then incubated with primary antibodies (anti-Ddi1) overnight at 4 °C, followed by incubation with fluorophore-conjugated secondary antibodies (1:100,000 in 3% BSA). DAPI was added and incubated for 20 min. Thin blood smears of the stained cells were made on microscope slides and mounted with cover slips. The slides were imaged using a Nikon Eclipse Ti-E microscope (Nikon Corporation, Tokyo, Japan). Images were processed using the NIS-Elements AR (4.40 version) software. Pearson correlation coefficient (PCC) values were calculated from the fluorescence intensities to indicate the degree of spatial co-localization between the *Pf*Ddi1 and DAPI. For 3D reconstruction, we used Imaris ×64 version 6.7 (Bitplane, Belfast, UK).

### 4.11. In Situ DNA Fragmentation (TUNEL) Assay

Treated or solvent alone cells (trophozoite-rich) were fixed and permeabilized as described above. DNA fragmentation was assessed by TUNEL using In Situ Cell Death Detection Kit, TMR Red (Roche Applied Science, Mannheim, Germany), following the manufacturer’s instructions. Briefly, the permeabilized cells were incubated with TdT enzyme and fluorescein-12-dUTP for 1 h at 37 °C, followed by incubation with DAPI for 20 min and then washed in 1 × PBS. Thin blood smears of the labeled parasite cells were made on microscope slides and then imaged as described above. The percentage (average) of cells with DNA breaks (TUNEL-positive cells) was estimated by counting the breaks (green fluorescence) against the total cells in several fields (*n* = 200).

### 4.12. Generation of Transgenic Yeast Cells

To know whether the *Pf*Ddi1 is a true yeast orthologue, we performed complementation studies in *S. cerevasie* yeast cells. We amplified deletion constructs using primers bearing the nourseothricin (NAT) selection marker (Supplementary Table S1). The genes were deleted by homologous recombination and the integration confirmed by PCR-based genotype analysis. On the other hand, the genes encoding full-length *Pf*Ddi1 were amplified from gDNA using primers as shown in supplementary Table S1. The PCR products were cloned into pGPD2 yeast expression vector at SpeI/XhoI site. The constructs were then transformed into the *S. cerevisiae* strain BY4741 by the lithium acetate method [50]. Selection of transformants were performed by plating over a synthetic complete (SC) medium lacking uracil. Deletions were confirmed by genomic DNA PCR with an appropriate set of primers (Supplementary Table S1).

### 4.13. Phenotypic Characterization

#### 4.13.1. Protein Secretion Assay and Growth Rate

The secretion assay was performed following the protocol described by [33]. Briefly, we inoculated a single colony from each strain into 5 mL synthetic complete (SC) medium (0.67% YNB with all amino acids but not uracil) in conical centrifuge tubes. The culture was incubated for 48 h at 30 °C in an orbital shaking incubator. We then pipetted 1 mL of culture into pre-weighed microcentrifuge tubes and separated the supernatant from pellet by centrifugation for 5 min at 13,000 rpm. The protein content in the supernatant was estimated using the Pierce BCA Protein Assay Kit (Thermo Scientific) with BSA as a standard. The cell pellet was dried at 100 °C and weighed. The assays were done in triplicates and the concentration was expressed as milligrams of protein secreted per milligram of dry cell weight.

#### 4.13.2. Treatment with (Genotoxic) Compounds

Yeast cells were grown to mid log phase and adjusted to 0.1 OD_600_. Serial dilutions were prepared and spotted on SC-based agar plates supplemented with hydroxyurea (50 µM), methylmethanesulfonate (0.05%), camptothecin (6 µg/mL), artemisinin (12 µM), chloroquine (3 mM), or lopinavir (50 µM). We used concentrations of the compounds that have been previously determined [51,52]. The plates were incubated at 30 °C for 48 h. For yeast growth curve assays, we followed the protocol as described by [53], with few modifications, in a liquid handling system (Tecan, Austria). Briefly, we inoculated a 96-well microplate with 5 µL of fresh midlog-phase cell cultures. Each well contained SC media supplemented with either of the compounds in a total volume of 0.2 mL. The cells were incubated for 24 h at 30 °C and the cell population was recorded at an interval of 30 min. All the samples were prepared in triplicates.

### 4.14. Statistical Analyses

We exported the data to Excel (Microsoft) and carried out statistical analyses and data representation using SPSS Statistics v16 or GraphPad Prism v6.0. Nonlinear regression analysis was used to determine the enzyme kinetic constants (K*_m_* and V_max_). The drug binding affinities (K_D_ values) were calculated after analysis of the association and dissociation from a 1:1 binding model. The results (bars) represent means ± standard error. The authors declare that they have no conflicts of interest with the contents of this article.

## 5. Conclusions

In conclusion, here we show that *Pf*Ddi1, an essential *P. falciparum* protein, is an active A_2_ family aspartic protease, with inherent abilities to degrade polyubiquitinated proteins. *Pf*Ddi1 is a true orthologue of *Sc*Ddi1 and might be involved in DNA damage repair strategies by the parasites. We further show that artemisinin, a first line drug against *P. falciparum* malaria, induces protein damage and might be inhibiting tidying up by blocking the activity of *Pf*Ddi1, a unique ubiquitin-proteasome retropepsin. These results thus provide new insight into the promiscuous actions of artemisinin and pave the way for potential development of new antimalarial drugs targeting *Pf*Ddi1.

## Data Availability

The data presented in this study are contained in this article.

## Supplementary Materials

The following are available online at https://www.mdpi.com/article/10.3390/pathogens10111465/s1. Figure S1: Expression and purification of full length *Pf*Ddi1 gene. Figure S2: LC-MS/MS proteome analysis of purified *Pf*Ddi1 fragments. Figure S3: Hydrolysis of substrates by the recombinant *Pf*Ddi1 enzyme. Figure S4: Artemisinin treatment leads to increased recruitment of *Pf*Ddi1 into the nucleus. Figure S5: Microscopic analysis of parasite morphologies under artemisinin treatment. Figure S6: SPR binding analysis of the compounds with heme detoxification protein (HDP). Figure S7: Multiple sequence analysis of the *Pf*Ddi1 gene. Table S1: List of primer sequences.
