# Supplementary material for: Artemisinin Binds and Inhibits the Activity of Plasmodium falciparum Ddi1, a Retroviral Aspartyl Protease
Source: Pathogens. 2021 Nov 11;10(11):1465. doi: 10.3390/pathogens10111465 (PMC8621276; doi:10.3390/pathogens10111465)
Supplement: Supplementary file 1 [file pathogens-10-01465-s001.zip › pathogens-1419373-supplementary.pdf]

Supplementary Figure S1: Expression and purification of full length *Pf*Ddi1 gene

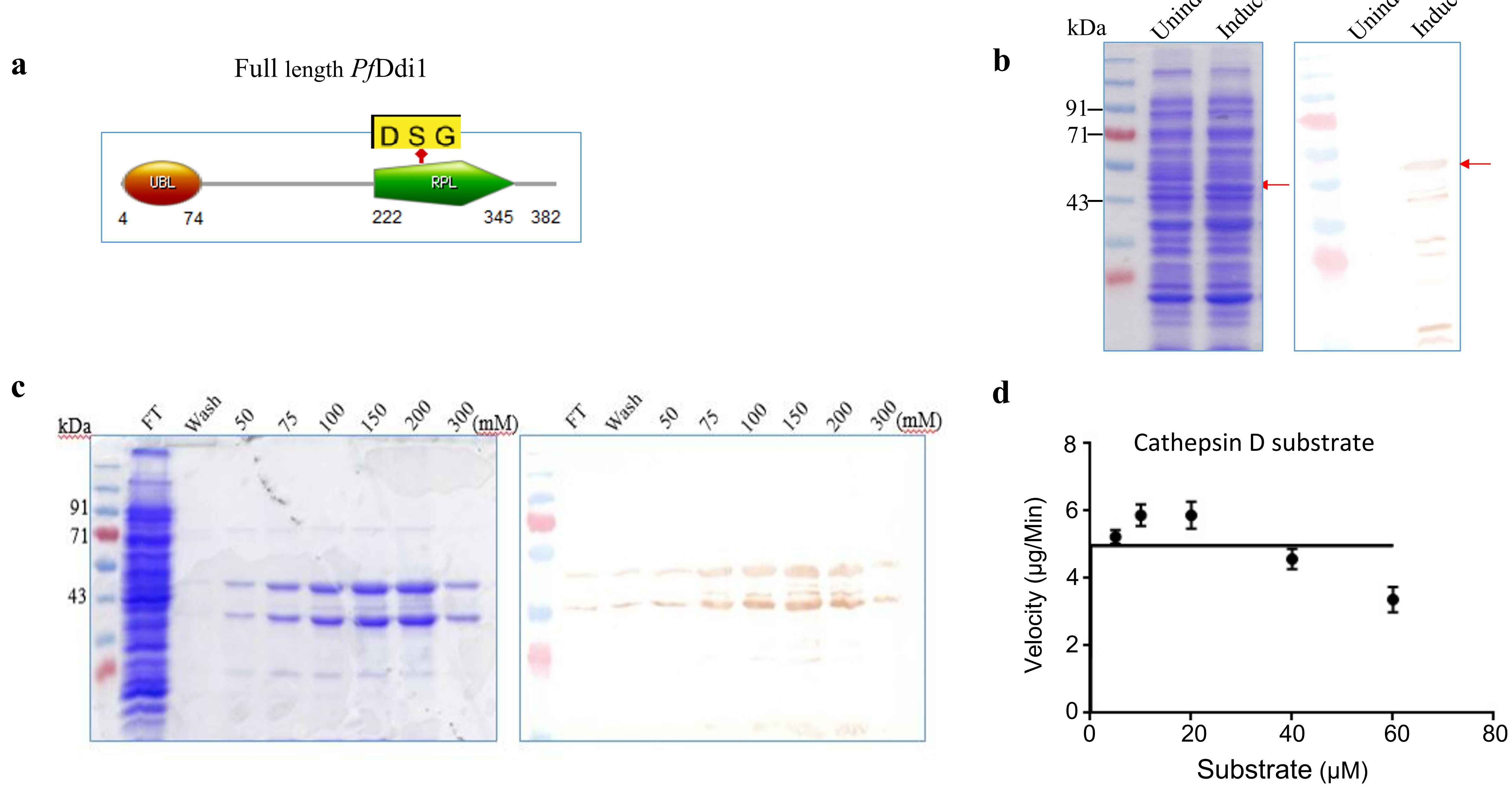

Supplementary Figure S2: LC-MS/MS proteome analysis of purified *Pf*Ddi1 fragments

a

~44 kDa *Pf*Ddi1

Accession	Description	Score	Coverage	# Proteins	# Unique Peptides	# Peptides	# PSMs	# AAs	MW [kDa]	calc. pI
PF3D7_1409300	organism=Plasmodium_falciparum_3D7 | product=DNA damage-inducible protein 1, putative (DDI1) | location=Pf3D7_14_v3:363092-364240(+) | length=382 | sequence_SO=chromosome | SO=protein_coding	2072.85	75.92	1	48	48	691	382	43.8	5.06

Sample Peptide reads

- FIYENIYKNEINNnLALAQEHFPEAFGVVFMLYIPVEInKNTVHAFVDSGAQSSImSK
- KISADImNDnVnNmSALNNILSTNNNVGNIGNIGNNLNNENVQNLLNNPAFK
- LQDEPLYNAIFSQNLEEIK
- FIYENIYKNEINnnLALAQEHFPEAFGVVFMLYIPVEInK
- FIYENIYKNEINnnLALAQEHFPEAFGVVFMLYIPVEInKNTVHAFVDSGAQSSIMSK

b

~34 kDa *Pf*Ddi1

Accession	Description	Score	Coverage	# Proteins	# Unique Peptides	# Peptides	# PSMs	# AAs	MW [kDa]	calc. pI
PF3D7_1409300	organism=Plasmodium_falciparum_3D7 | product=DNA damage-inducible protein 1, putative (DDI1) | location=Pf3D7_14_v3:363092-364240(+) | length=382 | sequence_SO=chromosome | SO=protein_coding	2200.58	73.56	1	46	46	700	382	43.8	5.06

Sample Peptide reads

- LQDEPLYNAIFSQNLEEIK
- LQDEPLYnAIFSQNLEEIK
- FIYENIYKNEINNNLALAqEHFPEAFGVVFMLYIPVEInKnTVHAFVDSGAQSSImSK
- FIYENIYKnEInNNLALAQEHFPEAFGVVFMLYIPVEINK
- KISADIMNDNVNNmSALNNILSTNNNVGNIGNIGNNLNNEnVQNLLNNPAFK

Supplementary Figure S3: Hydrolysis of substrates by the recombinant *Pf*Ddi1 enzyme

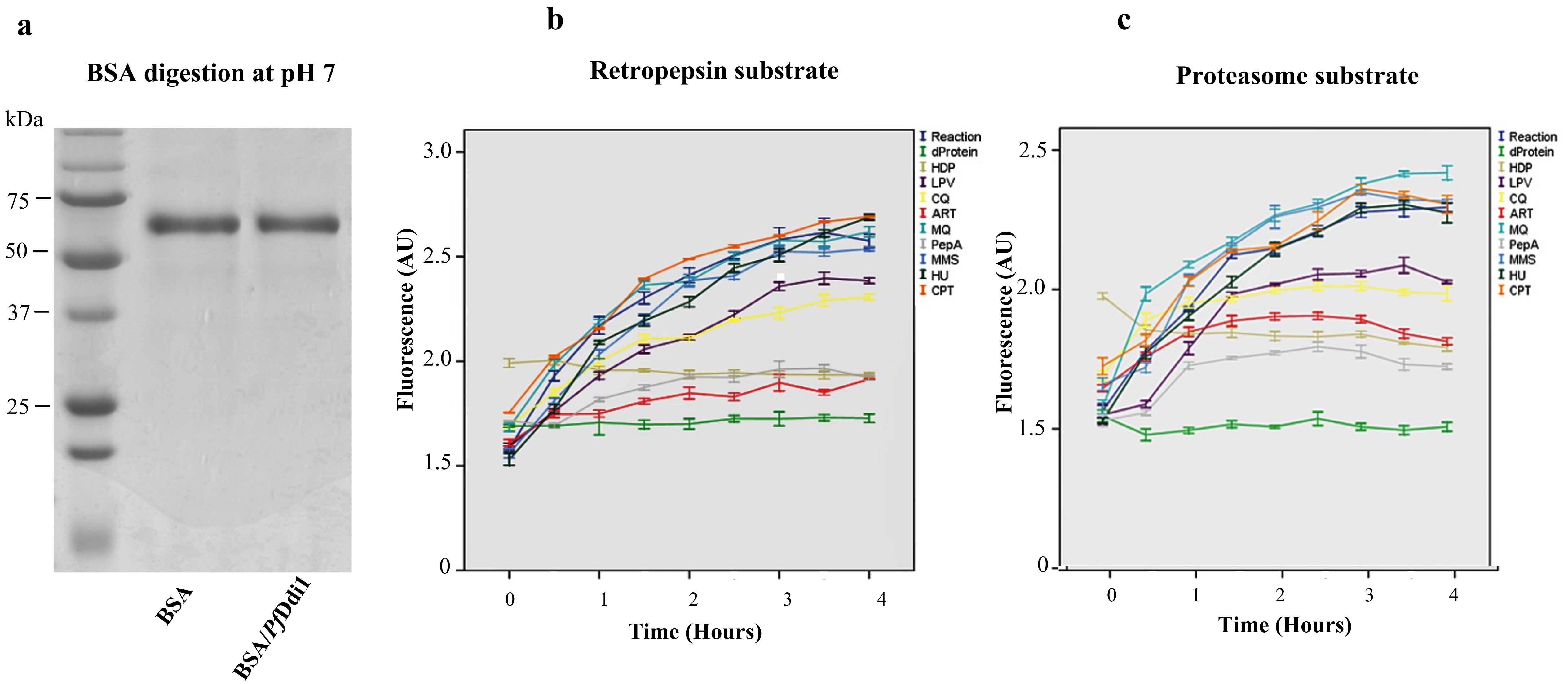

Supplementary Figure S4: Artemisinin treatment leads to increased recruitment of *Pf*Ddi1 into the nucleus

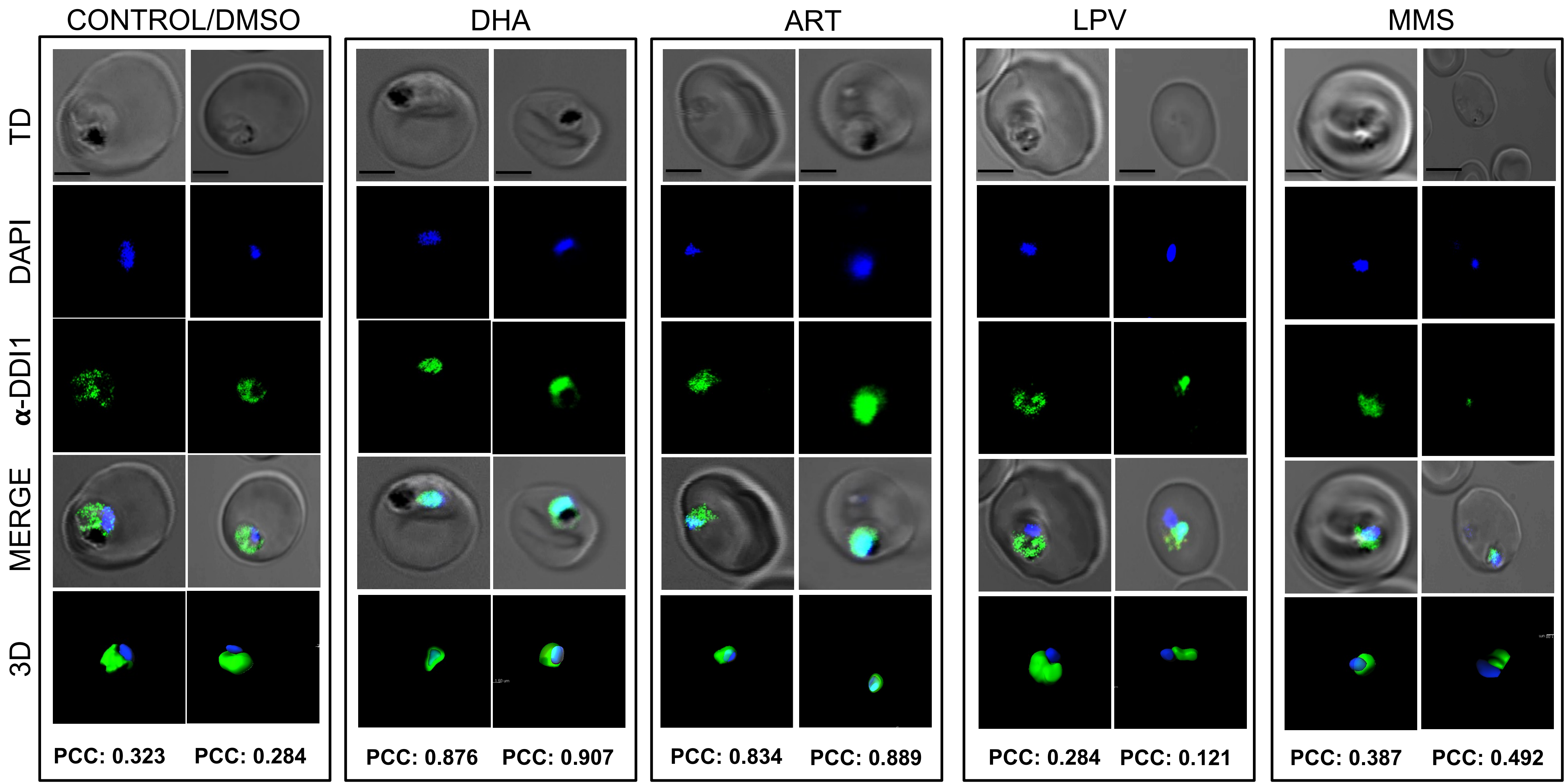

Supplementary Figure S5: Microscopic analysis of parasite morphologies under artemisinin treatment

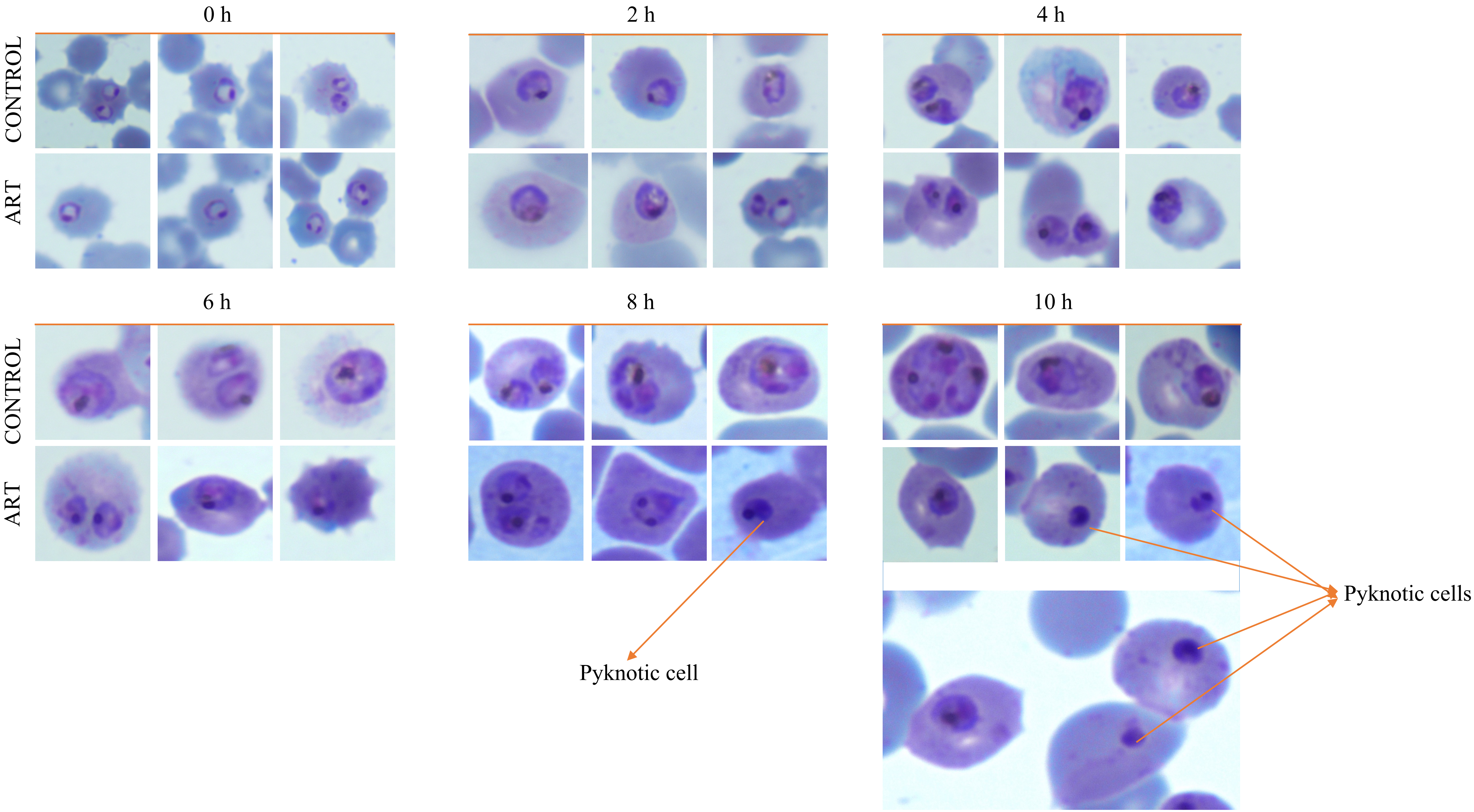

Supplementary Figure S6: SPR binding analysis of the compounds with heme detoxification protein (HDP)

a

ART

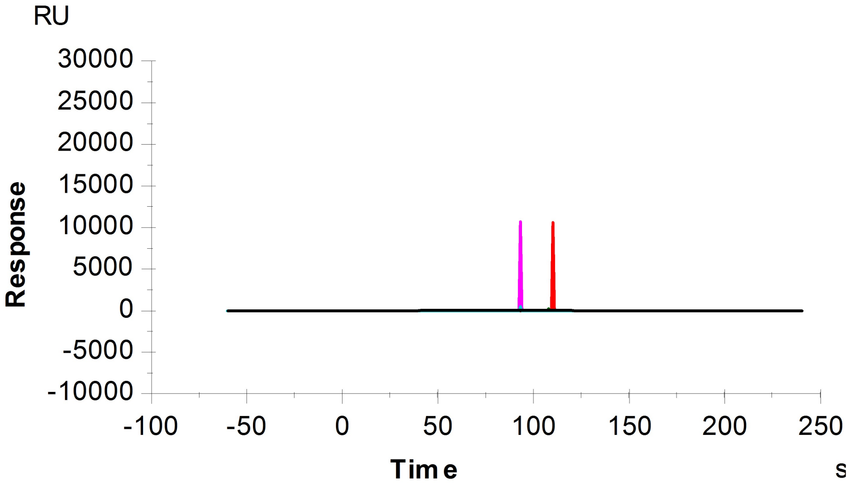

b

MMS

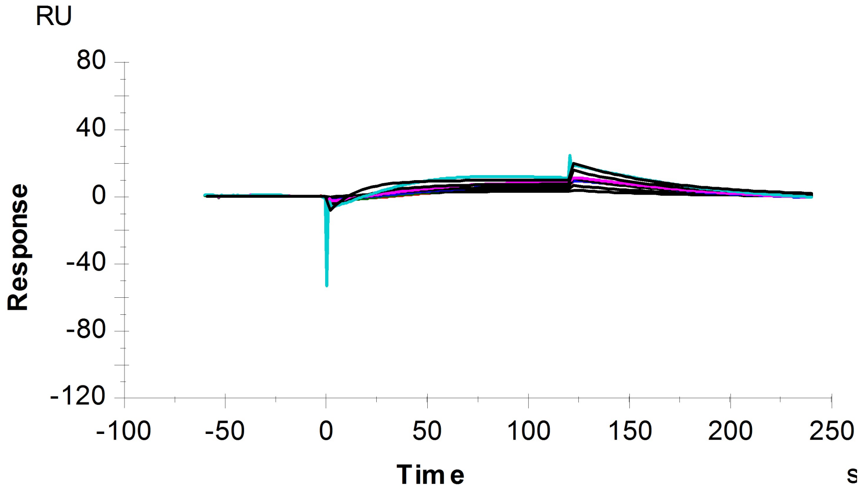

c

LPV

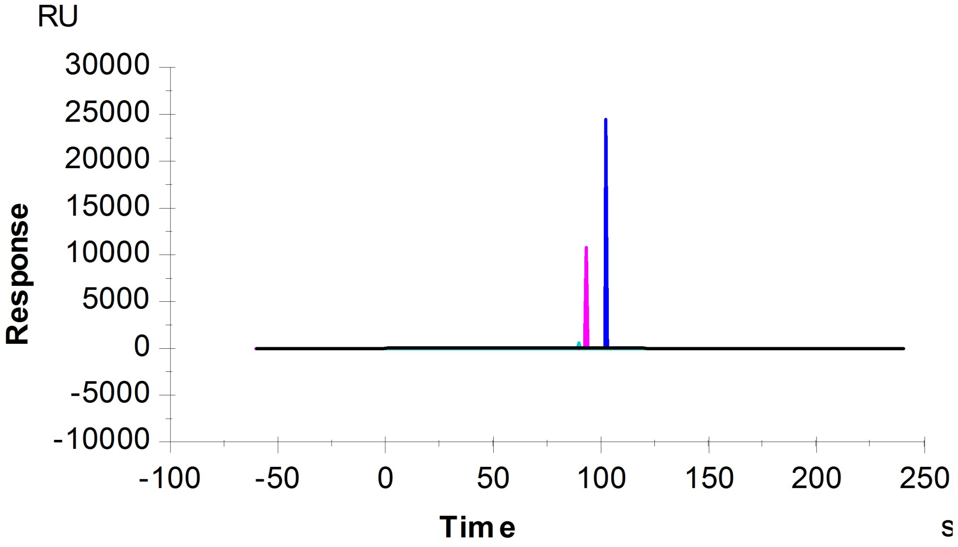

d

Compounds	Binding Energy (kcal/mol)
	Site Specific Docking
Artemisinin	-5.81
lopinavir	-1.43
Methyl	-2.67

Supplementary Figure S7: Multiple sequence analysis of the *Pf*Ddi1 gene

a

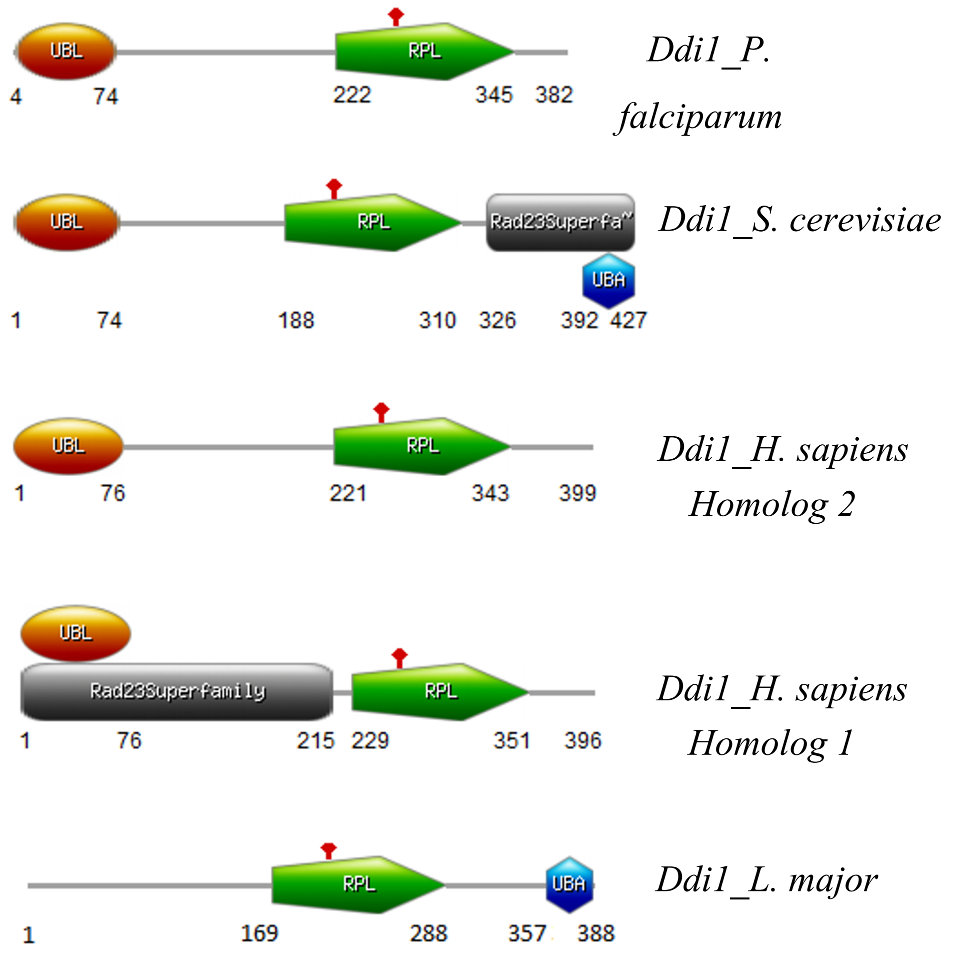

b

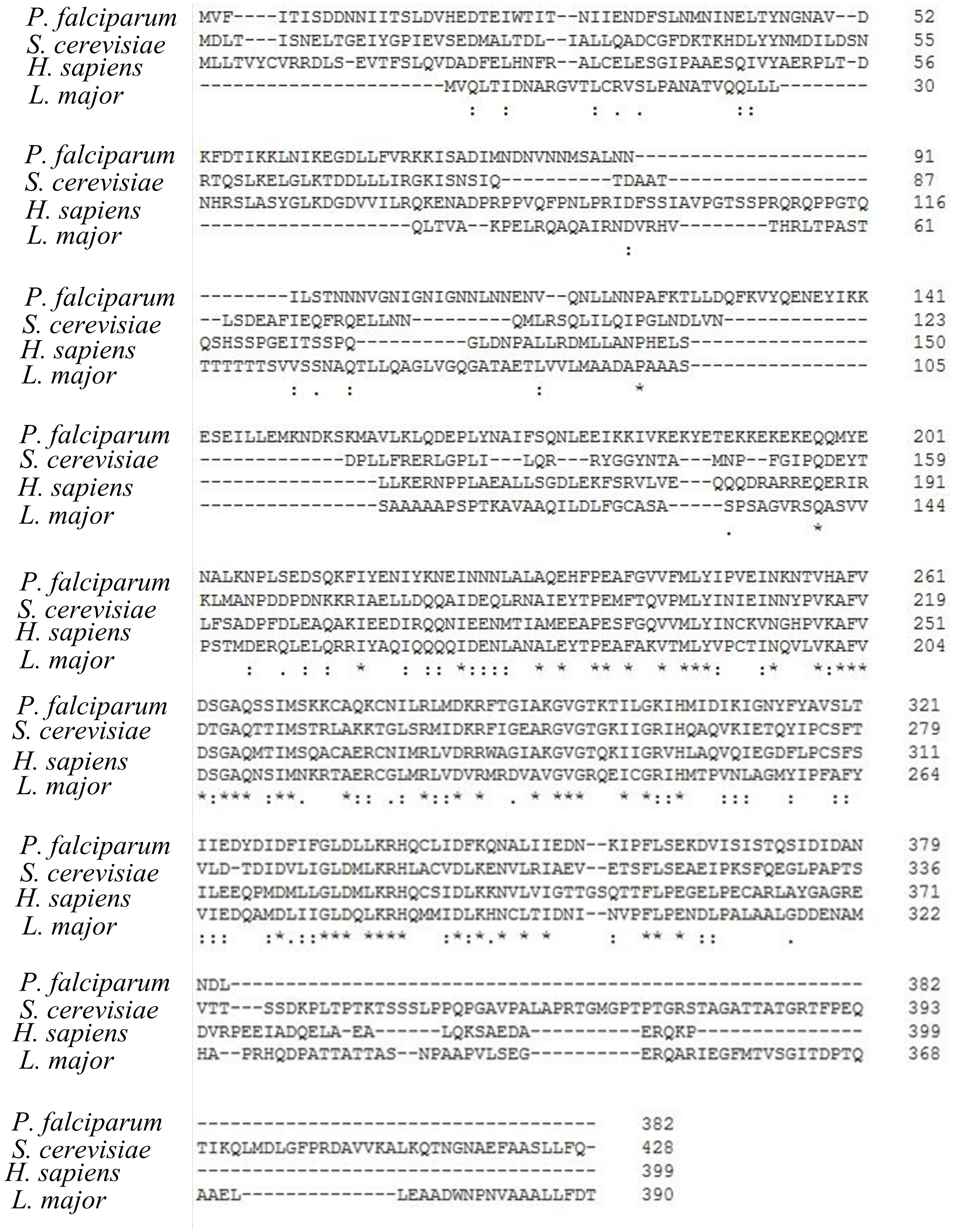

Supplementary Table S1: List of primer sequences

Deletion and confirmation primers		
Primer name	Sequence (5'-3')	
Ddi1_DEL_F	AGAAAGTATACTTAACGTAGTTAACAAAGTACATACCAAACATAACAGCAAAAATATACGTAAAGCCTTCCG[illegible]	
Ddi1_DEL_R	CTTATACTTATCTATTTGTGTTATGGGCTACATACGTAGAGGCCGATCACAATATCAGTGGTTGCTGCGGCCG[illegible]	
Ddi1_CON_1	GAGCAACCTCTAGAGGTCGAGAC	
Ddi1_CON_2	CCTACCAGGGGCCAAGTACCACC	
Ddi1_CON_3	ACTAGACTAATGGCCAACCCCGATG	COMMON PRIMERS FOR DELETION GENOTYPE ANALYSIS
Ddi1_CON_4	GGTGGGTGTTAGGGGCTTATCAGAC	
Ddi1_CON_5	TGCGCACGTCAAGACTGTCAAGG	
Ddi1_CON_6	TGTGAATGCTGGTCGCTATACTGC	
Cloning Primers in pGPD2		
Ddi1_*Sc*_F	CGCGAACTAGTATGGATTTAACAATTTCAAACG	
Ddi1_*Sc*_R	CGCGACTCGAGTCATTGGAAAAGGAGGGATGCAGC	
Ddi1_*Pf*_F	CGCGAACTAGTATGGTTTTTATTACAATATCAGAC	
Ddi1_*Pf*_R	CGCGACTCGAGTTATAAATCATTGTTTGCATCAATGTC	
